# Upregulation of neuronal zinc finger protein A20 expression is required for electroacupuncture to attenuate the cerebral inflammatory injury mediated by the nuclear factor-kB signaling pathway in cerebral ischemia/reperfusion rats

**DOI:** 10.1186/s12974-016-0731-3

**Published:** 2016-10-03

**Authors:** Jian Zhan, Wenyi Qin, Ying Zhang, Jing Jiang, Hongmei Ma, Qiongli Li, Yong Luo

**Affiliations:** 1 Department of Neurology, The First Affiliated Hospital of Chongqing Medical University, Chongqing, 400016 China; 2 Chongqing Key Laboratory of Neurology, 1 Youyi Road, Yuzhong District, Chongqing, 400016 China; 3 Department of Neurology, The Affiliated Hospital of Zunyi Medical College, Zunyi, Guizhou Province 563000 China; 4 Department of Integrated Chinese and Western Medicine, The First Affiliated Hospital of Chongqing Medical University, Chongqing, 400016 China

**Keywords:** Zinc finger protein A20, Cerebral ischemia, Neuroinflammation, Electroacupuncture, NF-kB signaling pathway

## Abstract

**Background:**

Zinc finger protein A20 (tumor necrosis factor alpha-induced protein 3) functions as a potent negative feedback inhibitor of the nuclear factor-kB (NF-kB) signaling. It exerts these effects by interrupting the activation of IkB kinase beta (IKKβ), the most critical kinase in upstream of NF-kB, and thereby controlling inflammatory homeostasis. We reported previously that electroacupuncture (EA) could effectively suppress IKKβ activation. However, the mechanism underlying these effects was unclear. Therefore, the current study further explored the effects of EA on A20 expression in rat brain and investigated the possible mechanism of A20 in anti-neuroinflammation mediated by EA using transient middle cerebral artery occlusion (MCAO) rats.

**Methods:**

Rats were treated with EA at the “Baihui (GV20),” “Hegu (L14),” and “Taichong (Liv3)” acupoints once a day starting 2 h after focal cerebral ischemia. The spatiotemporal expression of A20, neurobehavioral scores, infarction volumes, cytokine levels, glial cell activation, and the NF-kB signaling were assessed at the indicated time points. A20 gene interference (overexpression and silencing) was used to investigate the role of A20 in mediating the neuroprotective effects of EA and in regulating the interaction between neuronal and glial cells by suppressing neuronal NF-kB signaling during cerebral ischemia/reperfusion-induced neuroinflammation.

**Results:**

EA treatment increased A20 expression with an earlier peak and longer lasting upregulation. The upregulated A20 protein was predominantly located in neurons in the cortical zone of the ischemia/reperfusion. Furthermore, neuronal A20 cell counts were positively correlated with neurobehavioral scores but negatively correlated with infarct volume, the accumulation of pro-inflammatory cytokines, and glial cell activation. Moreover, the effects of EA on improving the neurological outcome and suppressing neuroinflammation in the brain were reversed by A20 silencing. Finally, A20 silencing also suppressed the ability of EA to inhibit neuronal NF-kB signaling pathway.

**Conclusions:**

Ischemia/reperfusion cortical neurons in MCAO rats are the main cell types that express A20, and there is a correlation between A20 expression and the suppression of neuroinflammation and the resulting neuroprotective effects. EA upregulated neuronal A20 expression, which played an essential role in the anti-inflammatory effects of EA by suppressing the neuronal NF-kB signaling pathway in the brains of MCAO rats.

## Background

Stroke is one of the leading causes of death and disability in the USA [1] and China [2], and most cases are caused by ischemic stroke in the area supplied by the middle cerebral artery [3]. However, the complex pathological mechanisms of ischemic/reperfusion injury in ischemic stroke have hindered the development of clinically effective treatments. Thus, there is an increasing need to seek effective intervention targets. In addition to the direct damage caused by ischemia and hypoxia after middle cerebral artery occlusion, excitotoxicity, calcium overload, oxidative stress, apoptosis, autophagy, and neuroinflammation are secondary injury mechanisms. In particular, induction of neuroinflammation which involves the interaction between neurons and glial cells could mobilize the brain tissue to generate cascade amplification effects of pathological process by crosstalk of multiple cytokines and signaling pathways [4]. Nuclear transcription factor kappa B (NF-kB) controls the initiation and development of the underlying inflammatory reaction. In the resting state, the p65/p50 heterodimeric and major form of NF-kB is mainly held in the cytoplasm bound to NF-kB inhibitor protein alpha (IkBα). Shortly after ischemia, the phosphorylated and activation of IkB kinase beta (IKKβ), the most critical kinase upstream of NF-kB, results in the phosphorylation and proteolysis of IkBα, which allows the p65/p50 dimer to translocate to the nucleus and promote the expression of pro-inflammatory cytokines (such as tumor necrosis factor alpha (TNF-α) and interleukin-1 beta (IL-1β). In the early stage of cerebral ischemia/reperfusion, this positive feedback cascade amplification loop occurs in neurons and glial cells that leads to an excessive inflammatory response in the brain and causes serious damage [5–12] including breakdown of the blood-brain barrier and brain edema, which is the main cause of early death in patients after stroke [13, 14]. Therefore, blocking the NF-kB signaling pathway is an effective strategy for reducing inflammatory injury after stroke [15].

Zinc finger protein A20 (TNF-α induced protein-3, TNFAIP3), initially known as TNF-induced and NF-kB transcription-dependent inflammatory inhibitor, was first identified in 1990 [16, 17]. Subsequent studies revealed that A20 functions as both a ubiquitin ligase and a deubiquitinating enzyme [18]. These functions of A20 could interrupt the phosphorylation of IKKβ activated by TNF-α, IL-1β, and other multiple upstream inflammatory signals [19–22]. Therefore, A20 is believed to be a key negative feedback inhibitor of the NF-kB signaling pathway and a potent endogenous protective factor against the inflammatory response [23–28] because of its ability to terminate the amplification of the positive feedback cascade. Previous animal experiments [29–33] showed that A20 overexpression could relieve inflammatory injury in the heart, liver, kidney, and other organs after ischemia/reperfusion. Another study [34] used DNA microarrays to identify that A20 was one of the potential anti-inflammation regulators for ischemic stroke. In addition, some studies [35, 36] have analyzed the expression of A20 in the brain after focal cerebral ischemia/reperfusion and assessed its underlying mechanism of action for regulating inflammation. However, there have been conflicting reports regarding the role of A20 in the central nervous system (CNS) [35, 37–39].

Electroacupuncture (EA) is a complementary combination therapy consisting of traditional acupuncture and electrotherapy. It is widely used worldwide, and particularly in China, for treating ischemic stroke and inflammatory diseases, and has achieved good results [40–47]. EA is also a candidate therapy recommended by the World Health Organization (WHO) to treat ischemic stroke. However, the precise mechanism of action and functional targets of EA in inflammatory damage after ischemia/reperfusion remain unclear, even though multiple studies [46, 48–50] have explored the ability of EA to regulate inflammatory injury. Our previous study revealed that EA could inhibit the NF-kB signaling pathway by suppressing IKKβ activation to reduce cerebral ischemic injury [51]. However, the specific mechanism behind these effects is yet to be elucidated.

A20 is a key factor that blocks IKK activation and inhibits the inflammatory reaction. Therefore, the current study further explored the effects of EA on A20 expression in the brain and investigated the possible mechanism by which EA regulates A20-mediated neuroprotection using transient middle cerebral artery occlusion (MCAO) rats. The role of A20 in focal cerebral ischemic/reperfusion-induced neuroinflammation and in regulating the interaction between neurons and glial cells (astrocytes and microglia) in neuroinflammation was also investigated.

## Methods

### Ethics statement

The experimental protocol used in this study was approved by the Ethics Committee for Animal Experimentation of Chongqing Medical University. All procedures were conducted according to the guidelines of the National Institutes for Animal Research.

### Animals

Specific pathogen-free (SPF) male Sprague-Dawley rats weighing 280–300 g were purchased from the Experimental Animal Center of Chongqing Medical University. They were housed under controlled conditions with 12-h light/dark cycles, a temperature of 22 ± 2 °C, and 60–70 % humidity for at least 1 week prior to surgery and treatment. All the rats were allowed for free access to a standard rodent diet and tap water.

### Lentivirus production and administration

All lentiviruses, including those containing A20 (LV-A20, 2 × 10^8^ transduction unit, TU/ml) and A20 shRNA (LV-shA20, 5 × 10^8^ transduction unit, TU/ml) for overexpressing and knocking down A20, respectively, and control (LV-ctrl) were purchased from Genechem (Shanghai, China). The viral dose to be administered was determined by rendering the 80 % cell infection rate in brain tissue. Ten days before surgery, the rats received an intracerebral ventricular injection of 5 μl of LV-A20, LV-shA20, or LV-ctrl at the calculated dose. The effects of gene interference on A20 overexpression or silencing were validated using real-time quantitative polymerase chain reaction (RT-qPCR) and western blotting.

### Intracerebral ventricular injection

The rats were anesthetized by intraperitoneally administering 3.5 % chloral hydrate (1 ml/100 g) and prostrated in a stereotaxic instrument (Stoelting, USA). A midline scalp incision was made from the overhead to expose the skull and bregma. According to stereotactic mapping combined with the rat size, a cranial hole located 1.3 mm lateral and 1.5 mm posterior to the bregma was drilled on the right hemisphere. A 10-μl Hamilton syringe (Hamilton Co., Reno, NV, USA) was slowly placed through the hole into the right lateral ventricle at a depth of 3.8 mm beneath the dural surface. Then, the lentivirus or vehicle was infused into the right lateral ventricle at a rate of 0.5 μl/min; the needle was held in place for an additional 5 min before withdrawal to prevent reflux.

### Focal cerebral ischemia/reperfusion

Focal cerebral ischemia/reperfusion was induced by MCAO using the intraluminal filament technique described in previous studies [51, 52]. Briefly, anesthetization was accomplished by administering 3.5 % chloral hydrate (1 ml/100 g) by intraperitoneal injection before surgery. The rats were fastened on a 37 °C thermostatic table, and the right common carotid artery and the right external carotid artery were exposed using a ventral midline neck incision. A 2.0 monofilament nylon suture (Ethicon Nylon Suture; Ethicon Inc., Osaka, Japan) with its tip rounded by heating was inserted through an arteriectomy in the external carotid artery and then advanced into the internal carotid artery ~18–20 mm distal from the carotid bifurcation until mild resistance indicated occlusion of the origin of the anterior cerebral artery and the middle cerebral artery. Reperfusion was accomplished by withdrawing the suture after 2 h of ischemia. The incision was then sutured and sterilized. Furthermore, regional cerebral blood flow (rCBF) was monitored using a disposable microtip fiber optic probe (diameter 0.5 mm) that was attached to the skull through a perforation and connected to a laser Doppler computerized main unit (PeriFlux 5000, Perimed AB, Sweden). The MCAO was considered adequate if the rCBF showed a sharp drop to 20 % and recovered to >80 % of baseline (pre-ischemia) level; the animals that did not meet this requirement were excluded from the analysis.

Body temperature was monitored in all animals. The rectal temperature was maintained at 38 ± 0.5 °C throughout the experiment using a rectal thermistor probe and a thermostatically regulated heating lamp placed above the body of the animal. The intrastriatal temperature was monitored and held at 36.5 ± 0.5 °C by manipulating the height of a small high-intensity lamp placed above the head during ischemia [53, 54].

### Electroacupuncture treatment (EA)

EA treatment was performed as described previously [51]. Briefly, the rats were anesthetized by the intraperitoneal injection of 3.5 % chloral hydrate (1 ml/100 g). According to the Experimental Animals Meridians Mapping, the “Baihui (GV 20),” “Hegu (L14),” and “Taichong (Liv3)” acupoints, which are located at the intersection of the sagittal midline and the line between the ears, the radial side of the left second metacarpal midpoint, and the dent between the first and second left metatarsal, respectively, were stimulated at an intensity of 1 mA and a frequency of 20 Hz for 5 min followed by 2 Hz for 30 min using a G6805-2 EA Instrument (Model no. 227033; Xinsheng Ltd., Qingdao, China). The rats were maintained on the 37 °C thermostatic table. EA treatment was given once daily until the rats were sacrificed; the first treatment began as the nylon monofilament was withdrawn.

### Neurobehavioral evaluation

Neurobehavioral evaluations were performed at the indicated time points (6, 12, 24, 48, and 72 h) by an investigator who was blind to the animal grouping. The 18-point system described by Garcia et al. [55] was used (with modifications) to assess the neurologic deficit.

### Infarct volume assessment

The infarct volume was assessed using 2,3,5-triphenyltetrazolium chloride (TTC, Sigma-Aldrich, USA) staining. Briefly, the rats were decapitated immediately following euthanasia 72 h after reperfusion. The brains were rapidly harvested and frozen for 10 min at −20 °C. Then, consecutive 2-mm-thick coronal sections were sliced, beginning 5 mm from the anterior tip of the frontal lobe, and stained with 2 % TTC for 10 min at 37 °C. Images were then captured using a digital camera (Canon IXUS, Canon Co., Japan) connected to a computer to evaluate the infarct volume. Unstained areas were defined as infarct, and the volume was measured using image analysis software (ImageJ) by an investigator who was unaware of the experimental grouping. Infarction volume was presented as a percentage of the intact hemisphere.

### Western blotting

The ischemic/reperfusion area of rat brains supplied by the right middle cerebral artery was delimited as described previously [56].^,^Coronal tissue sections 4-mm-thick were prepared starting 5 mm from the anterior tip of the frontal lobe, and then homogenized in RIPA lysis buffer (no. P0013B, Beyotime, Shanghai, China) supplemented with 1 mM phenylmethylsulfonyl fluoride (PMSF, Beyotime, Shanghai, China) and additional phosphatase inhibitors for detecting phosphorylated proteins. The total protein was extracted on ice from supernatants after centrifugation at 12,000 rpm for 10 min. In addition, cytoplasmic/nuclear proteins were extracted using a Nuclear and Cytoplasmic Protein Extraction Kit (no. P0027, Beyotime, Shanghai, China). Western blotting was performed as described previously [51] using electrophoresis apparatus (Bio-Rad Co., USA). Briefly, 50 μg protein in each lane was separated by SDS-PAGE (Beyotime, Shanghai, China) and transferred to polyvinylidene fluoride (PVDF) membranes (Millipore Co., USA). After blocking with 5 % skim milk/TBST, the membranes were incubated with primary antibodies for 2 h. The following primary antibodies were used: anti-A20 rabbit monoclonal (ab92324, Abcam, USA, 1:500 dilution), anti-IKKβ rabbit monoclonal (ab124957, Abcam, 1:500), anti-phospho-IKKβ (Ser176/180) rabbit monoclonal (#2697, Cell Signaling Technology, USA, 1:500), anti-NF-kB p65 mouse monoclonal (#6956, Cell Signaling Technology, 1:800), anti-IkBα rabbit polyclonal (18220-1-AP, Proteintech™ Co., USA, 1:500), and anti-phospho-IkBα (Ser32) rabbit monoclonal (#2859, Cell Signaling Technology, 1:500). They were then incubated with the appropriate secondary horseradish peroxidase-conjugated goat anti-rabbit (Proteintech™, 1:2000) or goat anti-mouse antibodies (Proteintech™, 1:2000) for 2 h. Gel imaging apparatus (Vilber Lourmat fusion FX 7 Spectra, France) and analysis software (FUSION-CAPT, France) were used to scan and analyze the blots.

### Real-time quantitative polymerase chain reaction (RT-qPCR)

The total RNA was extracted from the same ischemic reperfusion areas of brain tissue used for western blotting using Trizol reagent according to the manufacturer’s instructions (TaKaRa Biotechnology Co, Japan). cDNA was synthesized using a PrimeScript™ RT reagent kit with gDNA Eraser (TaKaRa). RT-qPCR was conducted on an iQ5 Gradient Real-Time PCR detection system (Bio-Rad Co., USA) using SYBR Green (SYBR Premix Ex *Taq*™ II, TaKaRa) for fluorescent quantification. The following cycling conditions were used: 30 s at 95 °C followed by 40 cycles of 5 s at 95 °C and 30 s at 60 °C. The melting curve of each sample was analyzed to determine primer-target specificity.

The PCR results were quantified using the threshold cycle (Ct) method, and the relative mRNA expression of target genes including *tumor necrosis factor alpha-induced protein 3* (*A20*), *Bcl-2 related protein A1*α (*A1*), and *glial fibrillary acidic protein* (*GFAP*) was normalized to that of the housekeeping gene β-actin. The nucleotide sequences of the primers used are shown in Table 1.

**Table 1:** List of primers used for real-time PCR

Gene	Definition	Accession	Forward	Reverse
A20	Rattus norvegicus tumor necrosis factor, alpha-induced protein 3 (Tnfaip3), transcript variants 1, 2, 3, and 4	XM_001060914.5, XM_006222835.2, XM_006222836.2, XM_003748656.3	5′-GACCACGGCACGACTCACCT-3′	5′-GGACAGTTGGGCGTCTCACAT-3′
A1	Rattus norvegicus BCL2-related protein A1 (Bcl2a1)	NM_133416.1	5′-AAGCTTCCACAAGAGCAGATTG-3′	5′-CAGCCAGCCAGATTTAGGTTC-3′
GFAP	Rattus norvegicus glial fibrillary acidic protein (Gfap)	NM_017009.2	5′-AGTGGTATCGGTCCAAGTTTGC-3′	5′-GTTGGCGGCGATAGTCATTAG-3′
β-actin	Rattus norvegicus actin, beta (Actb),	NM_031144.3	5′-ACGGTCAGGTCATCACTATCG-3′	5′-GGCATAGAGGTCTTTACGGATG-3′

### Immunofluorescence labeling

Twenty-four hours after reperfusion, the rats were transcardially perfused with saline and 4 % paraformaldehyde at 4 °C under deep anesthesia. The brain was removed rapidly, fixed in 4 % paraformaldehyde for 48 h, fully dehydration in 30 % sucrose solution, and cut into 10-μm-thick continuous coronal brain slices beginning 5 mm from the anterior tip of the frontal lobe. Then, the sections were processed by permeating the membrane with 0.3 % Triton X-100 for 30 min and performing antigen retrieval using 0.01 M sodium citrate in the microwave for 25 min. Nonspecific antigens were then blocked with 5 % goat or donkey serum for 1 h at 37 °C.

Double staining immunofluorescence was used to determine the cell types in the rat brain that express A20 and demonstrate NF-kB p65 express in neuron. The sections were incubated with the following primary antibodies at 4 °C overnight: anti-NeuN mouse monoclonal (to label neurons; MAB377, Millipore Co., Germany, 1:100 dilution), anti-GFAP mouse monoclonal (to label astrocytes; BM0055, Boster, China, 1:100), anti-Iba-1 goat polyclonal (to label microglia (NB100-1028, Novus Co., USA, 1:50), anti-A20 rabbit monoclonal (ab92324, Abcam, 1:100), and anti-NF-kB p65 rabbit monoclonal (#8242, Cell Signaling Technology, 1:100). After washing, the sections were incubated with the following fluorescent secondary antibodies for 2 h at 37 °C: Alexa Fluor 594-conjugated goat anti-rabbit IgG (H+L) (SA00006-4, Proteintech, 1:200), Alexa Fluor 488-conjugated goat anti-mouse IgG (H+L) (SA00006-1, Proteintech, 1:200), Alexa Fluor 594-conjugated donkey anti-rabbit IgG (H+L) (SA00006-8, Proteintech, 1:200), and FITC-conjugated Affinipure donkey anti-goat IgG (H+L) (SA00003-3, Proteintech, 1:200).

To quantify the immunofluorescence-labeled A20 expression, distinct A20-positive cells were counted in the ischemic brain tissue and expressed as number per mm^2^ (the cell density converted from cell number in 9 × 10^4^ pixels/in.^2^ of images with ×200 magnification) based on the method described by Wang et al. [57] with some modifications. Five view fields in the ischemic cortex were selected randomly for each sample, and A20-positive cell counts were performed by an investigator who was blinded to the experimental groups. All images were collected using an immunofluorescence microscope (Axio Observer ZI, Carl Zeiss Micro-Imaging Co., German) and processed using image processing software (Adobe Photoshop CS5, USA).

### Immunohistochemistry (IHC)

IHC was performed to show NF-kB p65 nuclear translocation at 24 h after reperfusion. Briefly, the rat brains were fixed, dehydrated, and trimmed as the same method used in the immunofluorescence assay and embedded in paraffin for slicing into 5-μm-thick sections. After dewaxing and rehydrating, the sections were immersed in 0.01 M citrate buffer for antigen retrieval using the microwave for 25 min. Endogenous peroxides were blocked by 3 % H_2_O_2_ for 10 min and nonspecific antigens were then blocked with 5 % goat serum for 20 min at 37 °C. The sections were incubated with primary anti-NF-kB p65 rabbit monoclonal antibody (#8242, Cell Signaling Technology, 1:200) at 4 °C overnight, and in sham group, the primary antibody was replaced with PBS as negative control. The SABC kit (including biotinylated goat anti-rabbit secondary antibody and Strept-Avidin-Biotin Complex; SA1028, BOSTER, China) and 3,3′-diaminobenzidine tetrahydrochloride (DAB) peroxidase substrate (AR1022, BOSTER, China) were used to detect NF-kB p65 immunostain. Images were scanned and acquired using an OLYMPUS PM20 automatic microscope (Olympus, Tokyo, Japan).

### Enzyme-linked immunosorbent assay (ELISA)

The levels of TNF-α and IL-1β in brain homogenates were determined using ELISA (no. SEA133Ra 96T and SEA563Ra 96T, Cloud-Clone Corp, USA) following the manufacturer’s instructions.

### Statistical analysis

GraphPad Prism Version 5.0 was used for all statistical analyses. Neurological scores are presented as median (interquartile ranges) and were analyzed using Kruskal-Wallis tests followed by post hoc Dunn’s multiple comparison tests. The rest of the data are expressed as means ± SEMs. The effect of EA on A20 mRNA or protein levels was compared among groups using two-way ANOVA with Bonferroni post hoc tests. All other data were analyzed using one-way ANOVA followed by Tukey’s multiple comparison tests to analyze inter-group differences. Correlations between A20-positive cell counts and neurologic scores, infarct volume, TNF-α and IL-1β content, and the relative mRNA levels of A1 and GFAP were investigated using Pearson’s correlation coefficients. *P* < 0.05 was considered to indicate statistical significance. All statistical figures were also generated using GraphPad Prism Version 5.0.

## Results

### EA upregulates A20 expression following focal cerebral ischemia-reperfusion

**Fig. 1:**
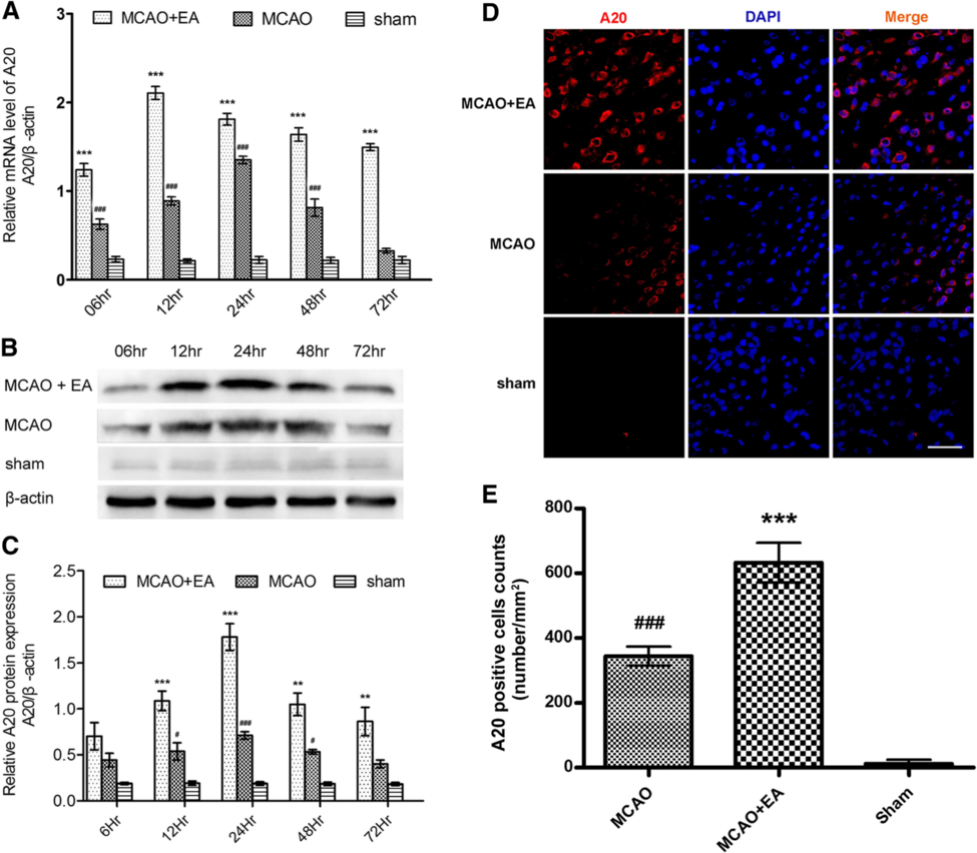
Effects of EA on A20 expression in the rat brain after I/R. **a** A20 mRNA levels in the rat brain were measured at the indicated time points using RT-qPCR. The graph shows the relative mRNA levels after normalization to the housekeeping gene β-actin. Data are presented as means ± SEM; *n* = 5 per time point per group. ***P < 0.001 vs. the MCAO group; ^###^ *P* < 0.001 vs. the sham group. **b** Western blots showing the expression of A20 in rat brains at the indicated time points. The housekeeping protein β-actin was used as a loading control. **c** Graph showing the relative A20 protein levels after normalized to β-actin. Data are presented as means ± SEM; *n* = 3 per time point per group. **P < 0.01, ***P < 0.001 vs. the MCAO group; ^#^ *P* < 0.05, ^###^ *P* < 0.001 vs. the sham group. **d** Immunofluorescence staining of A20 expression in the ischemia/reperfusion cortex of MCAO and MCAO + EA group rats 24 h after reperfusion and in the cortex of the sham group rats. *Scale bar* = 50 μm. **e** The A20-positive cell counts in the ischemia/reperfusion cortex of MCAO and MCAO + EA rats and the cortex of rats in the sham group. The A20-positive cell counts were expressed as number/in.^2^. Data are presented as means ± SEM; *n* = 5 per group. ^###^ *P* < 0.001 vs. the sham group; ***P < 0.001 vs. the MCAO group

The expression of A20 mRNA and protein in rat ischemia/reperfusion brain tissue was quantified using RT-qPCR and western blotting, respectively, at the indicated time points (6, 12, 24, 48, and 72 h after reperfusion) in three groups (MCAO, MCAO + EA, sham; *n* = 5 per group for RT-qPCR test and *n* = 3 per group for western blotting). Although beginning at 6 h after reperfusion, the simple ischemia/reperfusion insult induced higher *A20* mRNA transcription than that in the sham group (except at 72 h, all remaining time points *P* < 0.001; Fig. 1a), the peak occurred at 24 h after reperfusion and levels then dropped rapidly to normal comparable with the sham group at 72 h after reperfusion (*P* > 0.05; Fig. 1a). Moreover, *A20* mRNA levels were increased significantly further in the MCAO + EA group compared with the MCAO group (each time point *P* < 0.001; Fig. 1a), demonstrating earlier peak expression at 12 h after reperfusion and levels still remained high at 72 h after reperfusion. Similarly, the amount of A20 protein in the ischemic area was increased significantly in the MCAO + EA group compared with the MCAO group at each time point (at 12 h *P* < 0.001, at 24 h *P* < 0.001, at 48 h *P* < 0.01, at 72 h *P* < 0.01, respectively; Fig. 1b, c). However, levels were only increased after 12, 24, and 48 h of reperfusion in the MCAO group compared with the sham group (*P* < 0.05, *P* < 0.001, *P* < 0.05, respectively; Fig. 1b, c).

The spatial distribution of A20 in the brain tissues of rats from the three groups (MCAO, MCAO + EA, and sham; *n* = 5 per group) was identified using immunofluorescence staining. There was little A20 expression in the brains of rats in the sham group (Fig. 1d). Twenty-four hours after reperfusion the number of A20 immunopositive cells was increased significantly in the brains of rats in the MCAO group compared with the sham group (*P* < 0.001; Fig. 1e). The positive cells were located mainly in the focal cerebral ischemia/reperfusion cortex (Fig. 1e). Moreover, the A20-positive cell count in the MCAO + EA group was increased significantly compared with the MCAO group (*P* < 0.001; Fig. 1d, e).

### A20 is expressed mainly in neurons in the focal cerebral ischemia-reperfusion area

**Fig. 2:**
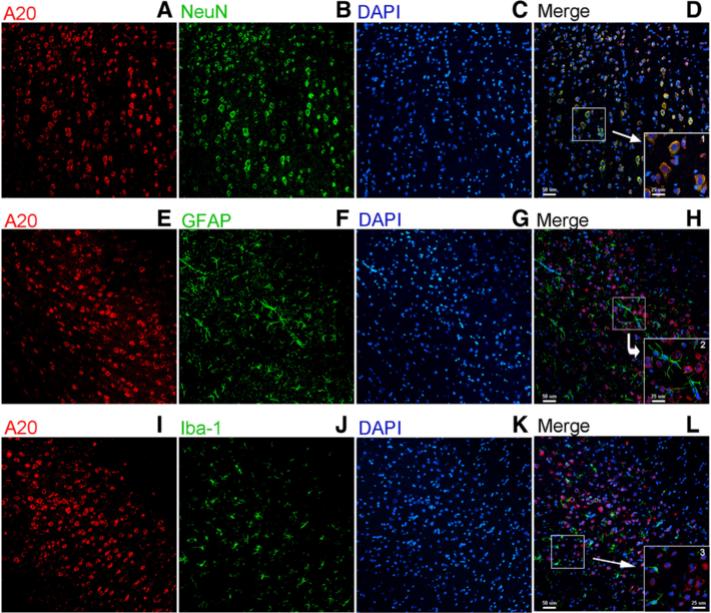
The spatial distribution and cell-type location of A20 in the rat brain after I/R. Brain sections of the ischemia/reperfusion cortex of rats in the MCAO + EA group 24 h after reperfusion. Sections were stained with double immunofluorescence labeling (**a**–**d**), which showed the upregulated expression of A20 protein (*red*) in neurons (*green*, identified using NeuN). **e**–**h** Very few of A20-positive (*red*) astrocytes (*green*, identified using GFAP) were observed. **i**–**l** A20 (*red*) was barely presented in microglial cells (*green*, identified using iba-I). *Scale bar* = 50 μm. *1*–*3* were the magnified parts from the merged images of **d**, **h**, **l** which more clearly showed the location relationship between A20 and neurons, astrocytes, and microglia, respectively. *Scale bar* = 25 μm

To further determine the main cellular localization of A20 in the rat brain, double immunofluorescence staining was performed to assess the localization of A20 in neurons, astrocytes, and microglia by co-staining for NeuN, GFAP, and ionized calcium binding adapter molecule 1 (Iba-1), respectively. Following 2 h of ischemia and 24 h of reperfusion, increased levels of A20 protein were found mainly in the neurons, whereas very little was observed in microglia and astrocytes (Fig. 2). This suggests that neurons play an important role in the inflammatory response in the brain after focal ischemia/reperfusion insult since A20 is a potent inflammatory inhibitor.

### A20 exerts neuroprotective effects

**Fig. 3:**
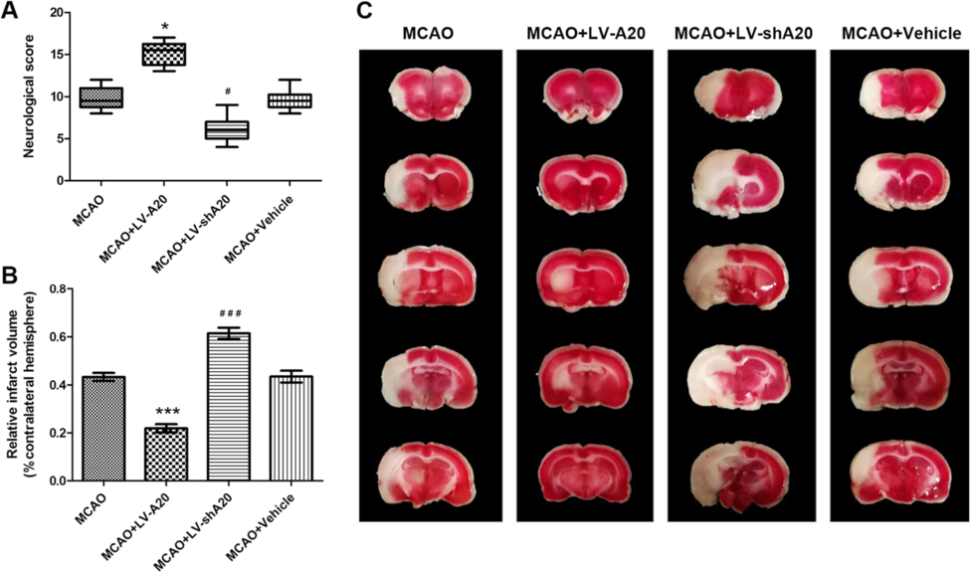
Neuroprotective effects of A20 on focal cerebral ischemia reperfusion injury. The neurological score and brain infarction volume were evaluated 72 h after reperfusion in rats in the four groups (MCAO, MCAO + LV-A20, MCAO + LV-shA20, and MCAO + vehicle). **a** Neurological scores are presented as median (interquartile range), *n* = 10 per group. *P < 0.05, ^#^ *P* < 0.05 vs. the MCAO group. **b** Brain infarct volume presented as a percentage of the intact hemisphere. Data are presented as means ± SEM, *n* = 10 per group. ***P < 0.001, ^###^ *P* < 0.001 vs. the MCAO group. **c** Images of cerebral infarction (*white*, infarct tissue; *red*, non-infarct tissue) were stained using 2,3,5-triphenyltetrazolium chloride in coronal sections of rat brains

To investigate the effects of A20 on cerebral ischemia/reperfusion injury, neurobehavioral evaluations were performed and the infarct volume was assessed using an 18-point neurologic deficit score and TTC staining, respectively. All analyses were performed in four groups (MCAO, MCAO + LV-A20, MCAO + LV-shA20, and MCAO + vehicle, *n* = 10 per group) at the indicated time points. Beginning after 6 h of reperfusion following 2 h of ischemia, there was no clear difference in neurological evaluation among groups (data not shown). However, 72 h after reperfusion, the rats in the MCAO + LV-A20 group exhibited far more notable mitigation of their neurologic dysfunction compared with those in the MCAO group (*P* < 0.05; Fig. 3a). In contrast, that was very little improvement in rats in the MCAO + LV-shA20 group compared with the MCAO group (*P* < 0.05; Fig. 3a). There was also no significant difference in neurologic score between the MCAO and MCAO + vehicle groups (*P* > 0.05; Fig. 3a).

Consistent with this, rats in the MCAO + LV-A20 group exhibited smaller brain infarct volumes compared with those in the MCAO group 72 h after reperfusion (*P* < 0.001, Fig. 3b, c). In addition, the infarct volume was larger in the MCAO + LV-shA20 group than the MCAO group (*P* < 0.001; Fig. 3b, c). The mean infarct volume was similar in the MCAO + vehicle and MCAO groups (*P* > 0.05; Fig. 3b, c). Therefore, these data suggest that the stroke outcome could be improved by the overexpression of A20 but exacerbated by A20 deficiency.

### A20 silencing largely prevents the neuroprotective effects of EA

Next, studies were performed to investigate whether the increased levels of A20 were essential for the therapeutic effects of EA on post-ischemia injury. The effects of A20 silencing on EA-induced neuroprotection were also assessed by performing neurobehavioral evaluations and measuring infarct volume in four groups (MCAO, MCAO + EA, MCAO + EA + LV-shA20, MCAO + EA + vehicle, *n* = 10 per group) 72 h after reperfusion.

**Fig. 4:**
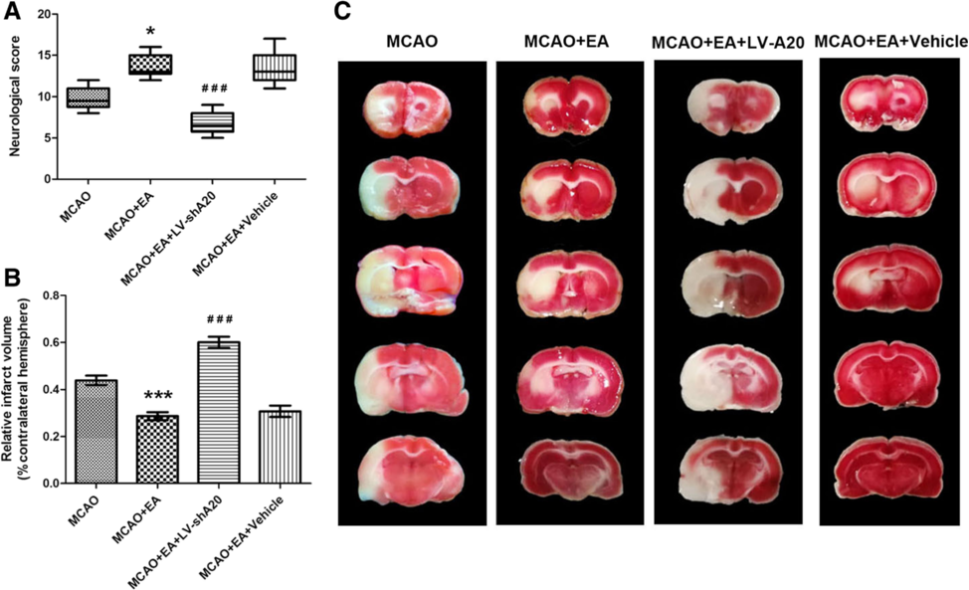
A20 silencing inhibits the neuroprotective effects of EA on focal cerebral ischemia reperfusion injury. Neurological score and brain infarction volume were evaluated 72 h after reperfusion in rats in the four groups (MCAO, MCAO + EA, MCAO + EA + LV-shA20, MCAO + EA + vehicle). **a** Neurological scores are presented as median (interquartile range), *n* = 10 per group. *P < 0.05 vs. the MCAO group; ^###^ *P* < 0.001 vs. the MCAO + EA group. **b** Brain infarction volume presented as a percentage of the intact hemisphere. Data are presented as means ± SEM, *n* = 10 per group. ***P < 0.001 vs. the MCAO group; ^###^ *P* < 0.001 vs. the MCAO + EA group. **c** Images of cerebral infarction (*white*, infarct tissue; *red*, non-infarct tissue) were stained using 2,3,5-triphenyltetrazolium chloride in coronal sections of rat brains

Compared with the MCAO group, the neurologic function of rats in the MCAO + EA group was improved (*P* < 0.05; Fig. 4a) and the infarct volume was significantly smaller (*P* < 0.001; Fig. 4b, c). In contrast, the conditions were worsened in the rats in the MCAO + EA + LV-shA20 group, as evidenced by a poor neurologic deficit and larger infarction volume compared with the MCAO + EA group (*P* < 0.05, *P* < 0.001, respectively; Fig. 4a–c). Finally, the neurologic scores and infarct volume were similar in the MCAO + EA + vehicle and MCAO + EA groups (*P*  >  0.05; Fig. 4a–c).

### A20 silencing inhibits the anti-inflammatory effects of EA

The effects of A20 silencing on the anti-inflammatory effects of EA were investigated in five groups (MCAO, MCAO + EA, MCAO + EA + LV-shA20, MCAO + EA + vehicle, sham, *n* = 5 per group) 24 h after cerebral ischemia/reperfusion.

**Fig. 5:**
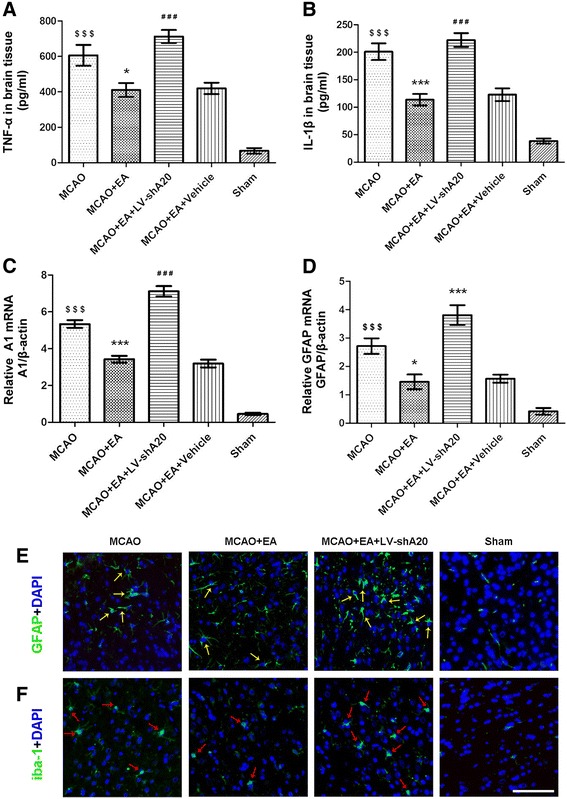
A20 silencing prevents the anti-inflammatory effects of EA after focal cerebral ischemia reperfusion. Experiments were performed 24 h after reperfusion in the ischemia/reperfusion cortex, *n* = 5 per group. **a** TNF-α and **b** IL-1β content was measured using ELISA. ^$$$^ *P* < 0.001 vs. the sham group; *P < 0.05, ***P < 0.001 vs. the MCAO group; ^###^ *P* < 0.001 vs. the MCAO + EA group. The relative mRNA levels of **c** *A1* and **d** *GFAP* were measured using RT-qPCR. ^$$$^ *P* < 0.001 vs. the sham group; ***P < 0.001, *P < 0.05 vs. the MCAO group; ^###^ *P* < 0.001 vs. the MCAO + EA group. The activated morphology of astrocytes and microglia was demonstrated using **e** GFAP and **f** Iba-1 immunofluorescence staining (*green*), respectively. *Yellow arrows* indicate reactive astrocytes with thick cell bodies, and *red arrows* indicate hypertrophied activated microglia, noted by their stout, dense appearance with shorter and thicker branched projections. *Scale bar* = 50 μm

Because the cytokines, TNF-α and IL-1β are the most important pro-inflammatory mediators after the onset of ischemia [58]. The levels of both cytokines were measured using ELISA to determine the scale of the inflammatory response in rat brain tissue. TNF-α and IL-1β levels in focal ischemia/reperfusion brain tissue were increased significantly in the MCAO group compared with the sham group (*P* < 0.001, *P* < 0.001, respectively; Fig. 5a, b) but were decreased significantly in the MCAO + EA group compared with the MCAO group (*P* < 0.05, *P* < 0.001, respectively; Fig. 5a, b). Moreover, an anti-inflammatory effects of EA were reduced significantly by silencing *A20* mRNA in the MCAO + EA + LV-shA20 group, since the brains exhibited higher TNF-α and IL-1β levels than those in the MCAO + EA group (*P* < 0.001, *P* < 0.001, respectively; Fig. 5a, b). There was no difference in TNF-α and IL-1β levels between the MCAO + EA and MCAO + EA + vehicle groups (*P* > 0.05, *P* > 0.05, respectively; Fig. 5a, b).

Innate immunocytes such as microglial [59] and astrocyte [60] play an essential role in the early stage of the inflammatory response after cerebral ischemia/reperfusion [61, 62]. Therefore, the activation of these innate immunocytes was detected by using RT-qPCR to measure the mRNA levels of *A1* and *GFAP*, which are markers of microglia and astrocyte [37] activation, respectively. Twenty-four hours after reperfusion, the relative levels of *A1* and *GFAP* were clearly elevated in the MCAO group compared with the sham group (*P* < 0.001, *P* < 0.001, respectively; Fig. 5c, d). However, levels were significantly lower in the MCAO + EA group compared with the MCAO group (*P* < 0.001, *P* < 0.05, respectively; Fig. 5c, d). Furthermore, compared with the MCAO + EA group, the relative levels of *A1* and *GFAP* were significantly higher in the MCAO + EA + LV-shA20 group (*P* < 0.001, *P* < 0.001, respectively; Fig. 5c, d). There was no difference between the MCAO + EA and MCAO + EA + vehicle groups (*P*  >  0.05, *P*  >  0.05, respectively; Fig. 5c, d).

The activation state of glial cells was investigated further by using immunofluorescence staining to assess morphological changes. Glial cell activation patterns appeared in the ischemia/reperfusion cortex of rats. Activated astrocytes show increased GFAP immunoreactivity and thickening of the cytoplasmic processes [63] (Fig. 5e). Activated microglia present with enlarged and brightened cell bodies together with shortened and thickened processes [64] (Fig. 5f). Glial cells in the brains of rats in the sham group were in the resting state, with a ramified morphology and delicate processes. Glial cell activation was the most extensive and evident in the cerebral ischemia/reperfusion area of the MCAO + EA + LV-shA20 group; however, it was inhibited by EA in the brains of rats in the MCAO + EA group. An intermediate phenotype was observed in the MCAO group (Fig. 5e, f). The morphological changes in the glial cells were consistent with the expression of *GFAP* and *A1* mRNA observed in the same groups.

### Correlation between neuronal A20 expression and neurologic outcome and inflammatory response after cerebral ischemia reperfusion

Next, the role of neuronal A20 expression in restraining the inflammatory response and attenuating neurologic dysfunction at the early stage of focal cerebral ischemia/reperfusion was investigated further. Neuronal A20-positive cells were counted in the focal cerebral ischemia/reperfusion area of rats in the four groups (MCAO, MCAO + EA, MCAO + EA + LV-shA20, MCAO + EA + vehicle, *n* = 5 per group). The results of neuronal A20-positive cell counts were paired randomly with those results in the correspondent groups from the above two A20-silencing experiments. Then, the correlation between neuronal A20-positive cell counts and neurologic scores, infarct volume, TNF-α and IL-1β content, and relative *A1* and *GFAP* mRNA levels was investigated, respectively (*n* = 20 pair, respectively), according to the method described by Wang et al. [65].

**Fig. 6:**
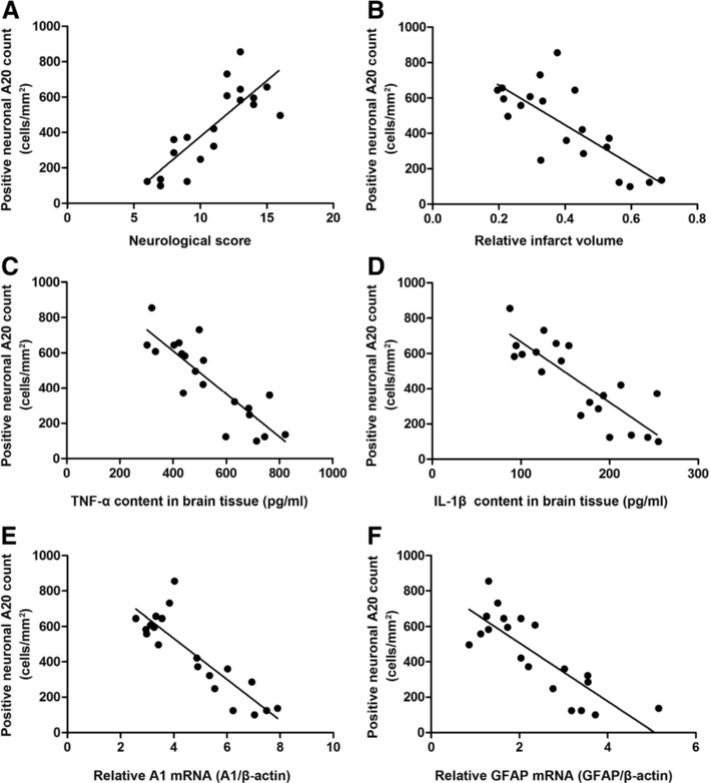
The correlations between A20-positive neuronal cell count and the inflammatory response and neurological outcome. **a** The relationship between the number of A20-positive neurons and neurological scores (*n* = 20 pairs, *r* = 0.8140, *P* < 0.0001). **b** The relationship between the number of A20-positive neurons and the relative infarct volume (*n* = 20 pairs, *r* = −0.7591, *P* < 0.0001). **c** The relationship between the number of A20-positive neurons and TNF-α content (*n* = 20 pairs, *r* = −0.8504, *P* < 0.0001). **d** The relationship between the number of A20-positive neurons and IL-1β content (*n* = 20 pairs, *r* = −0.8362, *P* < 0.0001). **e** The relationship between the number of A20-positive neurons and the relative *A1* mRNA level (*n* = 20 pairs, *r* = −0.8704, *P* < 0.0001). **f** The relationship between the number of A20-positive neurons and the relative *GFAP* mRNA level (*n* = 20 pairs, *r* = −0.8209, *P* < 0.0001)

There was a clear positive correlation between neuronal A20-positive cell counts and neurologic scores (*r* = 0.8140, *P* < 0.0001; Fig. 6a), and a significant negative correlation between neuronal A20-positive cell counts and infarct volume (*r* = −0.7591, *P* < 0.0001; Fig. 6b), TNF-α content (*r* = −0.8504, *P* < 0.0001; Fig. 6c), IL-1β content (*r* = −0.8362, *P* < 0.0001; Fig. 6d), relative *A1* mRNA levels (*r* = −0.8704, *P* < 0.0001; Fig. 6e), and relative *GFAP* mRNA levels (*r* = −0.8209, *P* < 0.0001, Fig. 6f).

### The upregulation of A20 is required for EA to inhibit neuronal NF-kB signaling

**Fig. 7:**
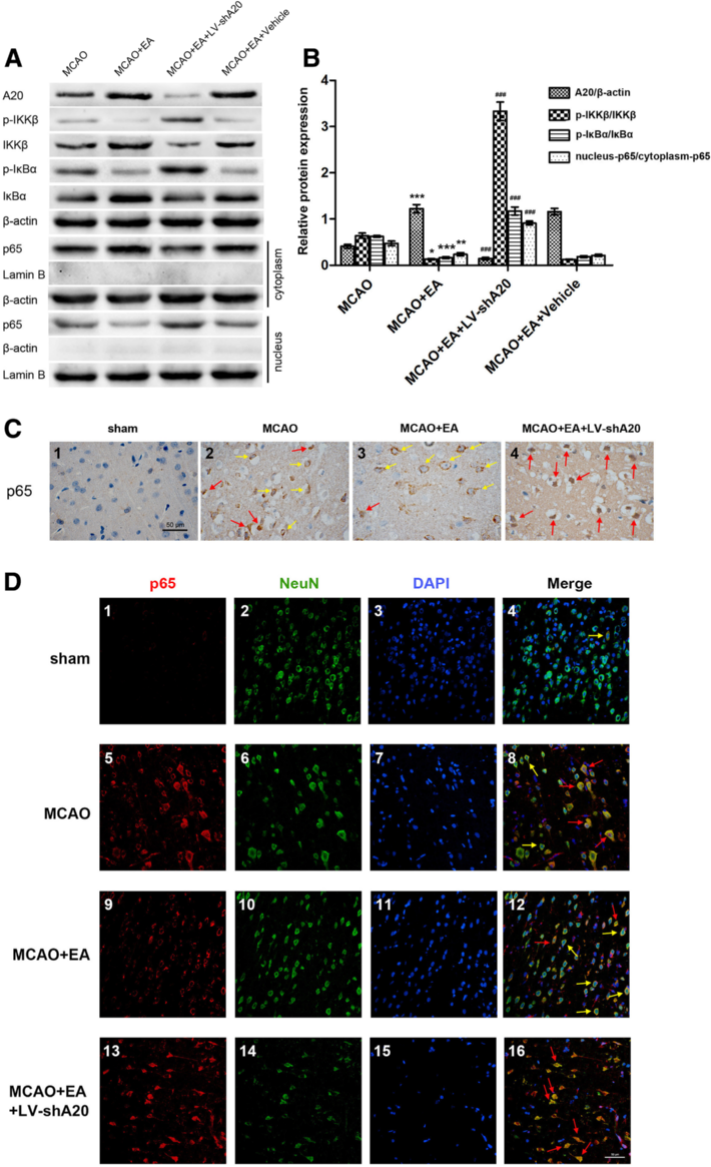
EA suppressed NF-kB signaling by upregulating A20. **a** Western blots showing the expression of A20, p-IKKβ, IKKβ, p-IkBα, IkBα, nuclear p65, and cytoplasmic p65 in ischemia/reperfusion cortical extracts 24 h after reperfusion. β-actin was used as the loading controls for total and cytoplasm proteins, and lamin B was used as the nuclear loading control. **b** The phosphorylation ratio of IKKβ and IkBα is presented as the p-IKKβ/IKKβ and p-IkBα/IkBα ratios, respectively. The nuclear translocation ratio of NF-kB p65 was indicated as nucleus-p65/cytoplasm-p65. *n* = 5 per group. *P < 0.05, **P < 0.01, ***P < 0.001 vs. the MCAO group; ^###^ *P* < 0.001 vs. the MCAO + EA group. **c** At 24 h after reperfusion, NF-kB p65 immunohistochemistry (*brown*) was performed to show NF-kB p65 nuclear translocation in the ischemia/reperfusion cortex of rats from the four groups (sham, MCAO, MCAO + EA, MCAO + EA + LV-shA20, *n* = 5 per group). *Yellow arrows* indicate NF-kB p65 mainly in cytoplasm, and *red arrows* indicate NF-kB p65 both in cytoplasm and nucleus. *Scale bar* = 50 μm. **d** Immunofluorescence staining was performed to demonstrate NF-kB p65 expression (*red*) in neurons (*green*) of ischemia/reperfusion cortex of rats from the four groups (sham, MCAO, MCAO + EA, MCAO + EA + LV-shA20, *n* = 5 per group) at reperfusion 24 h. *Yellow arrows* indicate NF-kB p65 mainly in cytoplasm, and *red arrows* indicate NF-kB p65 both in cytoplasm and nucleus. *Scale bar* = 50 μm

Next, we determined whether the increased neuronal A20 expression was essential for the therapeutic effects of EA on the post-ischemia inflammatory injury by inhibiting the NF-kB signal pathway. As shown in Fig. 7a, b, the rats were randomized into four groups (MCAO, MCAO + EA, MCAO + EA + LV-shA20, MCAO + EA + vehicle, *n* = 5 per group). Western blotting was used to measure A20, p-IKKβ, IKKβ, p-IkBα, IkBα, nuclear p65, and cytoplasmic p65 proteins in the focal ischemia/reperfusion area 24 h after reperfusion. The p-IKKβ/IKKβ and p-IkBα/IkBα, respectively, represent the phosphorylation ratio of IKKβ and IkBα, and nucleus-p65/cytoplasm-p65 represents the nuclear translocation ratio of NF-kB p65. All these ratios were used to comparatively assess the activity of the NF-kB signaling pathway between individual groups.

Twenty-four hours after reperfusion, there were increased A20 protein levels, a reduced phosphorylation ratio of IKKβ and IkBα, and suppressed nuclear translocation of NF-kB p65 in the MCAO + EA group compared with the MCAO group (*P* < 0.05, *P* < 0.05, *P* < 0.001, *P* < 0.01, respectively; Fig. 7b). In contrast, little A20 protein expression was detected in the MCAO + EA + LV-shA20 group, which also exhibited significantly elevated phosphorylation ratios of IKKβ and IkBα and the increased nuclear translocation of NF-kB p65 compared with the MCAO + EA group (*P* < 0.001, *P* < 0.001, *P* < 0.001, *P* < 0.001, respectively; Fig. 7b). These results suggest that neuronal A20 expression is an essential for the ability of EA to inhibit NF-kB signaling.

Immunohistochemistry was also performed to show NF-kB p65 nuclear translocation at 24 h after reperfusion. The rats were randomized into four groups (sham, MCAO, MCAO + EA, MCAO + EA + LV-shA20, *n* = 5 per group). As shown in Fig. 7c, immunostaining for NF-kB p65 was much stronger both in the cytoplasm and nucleus of large part of cells in the ischemia/reperfusion cortex of MCAO rats (Fig. 7c2), as compared to the faint staining observed in sham group (Fig. 7c1). Although MCAO + EA rats were also shown substantial of NF-kB p65 staining, most of which were restrained in the cytoplasm (Fig. 7c3). Nevertheless, significant NF-kB p65 staining was predominantly detected throughout those cells with shrank body in MCAO + EA + LV-shA20 group (Fig. 7c4). These results were in line with the Western blotting analysis above with the morphological characteristics of NF-kB p65 nuclear translocation. However, the morphological characteristics of specific cell type of NF-kB p65 nuclear translocation needed to be furthered.

Since A20 expression was mainly regulated in neurons by EA, its effect on neuronal NF-kB signaling and corresponding morphological changes of neurons were thereby exhibited by double immunofluorescence staining. The Rats were randomized into four groups (sham, MCAO, MCAO + EA, MCAO + EA + LV-shA20, *n* = 5 per group). Similar to immunohistochemistry results, only a small amount of NF-kB p65 were detected in the cytoplasm of individual neuron in the sham rat brains (Fig. 7d1–4) whereas, at reperfusion 24 h, sorts of brain cells had expressed NF-kB p65 in the ischemia/ reperfusion cortex of MCAO rats. Especially, a large number of neurons not only had significant NF-kB p65 immunofluorescence in the cytoplasm but also shown the positive staining in the nucleus, suggesting nuclear translocation. Moreover, these neurons arranged loosely and disconnectedly, with partial cell body representing swollen (Fig. 7d5–8). Contrarily, EA group has shown neuron arrangement basically kept closely and orderly in the ischemia/reperfusion cortex, and despite neuronal NF-kB p65 expression was also abundant, most of them were confined to the cytoplasm (Fig. 7d9–12). However, A20 silencing seriously damaged EA blocking NF-kB p65 nuclear translocation. The dense NF-kB p65 immunofluorescence staining was detected covering throughout cytoplasm and nucleus of nearly all neurons in the ischemia/reperfusion cortex of MCAO + EA + LV-shA20 rats. Accordingly, cell number in this area was decreased significantly, and the residual neurons arranged sparsely, with a shrank body and broken nucleus (Fig. 7d13–16). Technically, maybe the immunofluorescence was inferior to immunohistochemistry in demonstration effect of NF-kB p65 nuclear translocation, but it could better reveal the relationship between specific neuronal NF-kB signaling and corresponding morphological changes of neurons after ischemia/reperfusion injury. Thus, these results precisely indicated that A20 was involved in EA regulating neuronal NF-kB signaling and also effectively influenced the neuronal morphological characteristics, which could affect the neuron survival.

## Discussion

The inflammatory response induced by focal cerebral ischemia/reperfusion is a “double-edged sword” to the CNS, and the early excessive inflammatory response is a very important cause of secondary neuronal injury [66–74]. In this study, we established a rat model of focal cerebral ischemia/reperfusion and found that activated astrocytes and microglia act as inflammatory cells in brain tissue and that the accumulation of pro-inflammatory cytokines such as TNF-α and IL-1β contribute to the formation of cerebral infarction, deteriorated neurological function, and the induction of inflammatory brain damage. During this process, NF-kB signaling regulates the initiation and development of inflammation and activates a positive feedback that has an amplification effect. A20 is a key negative feedback inhibitor of the NF-kB signaling pathway. It regulates the dynamic balance of the inflammatory response and particularly prevents an excessive inflammatory response, chronic inflammation, and autoimmune diseases [24]. However, studies regarding the specific role of A20 in the regulation of the acute inflammatory responses induced by cerebral ischemia/reperfusion are in their infancy. Therefore, the current study explored the characteristics of A20 expression in the focal cerebral ischemia/reperfusion for the first time.

To date, A20 mRNA and protein have been detected in the heart, lung, liver, kidney, and other tissues and organs; however, its role in the bran is poorly understood. Although some studies [35, 36] found that A20 expression was upregulated in the rat brain after cerebral ischemia, suggesting that A20 may be involved in the injury mechanism, there was still no accurate description of the dynamic expression of A20. The current study revealed that there was little expression of A20 in the brain tissue of the sham group rats, suggesting that A20 may not be expressed in this tissue under normal physiological conditions. A20 mRNA and protein expression was induced rapidly by focal cerebral ischemia/reperfusion. The peak expression occurred 24 h after reperfusion, which was followed by a rapid drop; expression was restored to normal 72 h after reperfusion. It is unlikely that the upregulation of A20 in this manner would be timely enough and sufficient to inhibit inflammatory injury, the climax of which occurred just 24 h after focal cerebral ischemia/reperfusion. Several studies [29–33] have also confirmed this finding, while revealing the effects of A20 on depressing acute inflammation in the heart, liver, kidney, and other organs insulted by ischemia. This may be due to delayed inhibition of the negative feedback for the NF-kB signaling pathway, which led to an excessive and inappropriate inflammatory reaction in response to cerebral ischemia/reperfusion [75]. Therefore, this process needs to be controlled strictly. Furthermore, it is possible that improving the reaction rate of upregulating A20 and increasing A20 expression is a potential anti-inflammatory strategy. In particular, it is worth noting that the spatial distribution of A20 protein in the brain has not been directly exhibited by other study so far.

Pranski et al. [76] detected *A20* mRNA but not protein expression in the frontal cortex, striatum, hippocampus, and brain stem of the normal human brain. They speculated that, under normal physiological conditions, only very low levels of A20 protein synthesis occurs, whereas upregulation occurs under certain pathological conditions, consistent with the current study. In addition, they hypothesized that neurons may be the main cell type that expresses A20 based on the indirect evidence that some components of the A20 ubiquitin editing enzyme complex, such as RNF11 (RING finger protein 11), TAXIBP1 (Tax1 binding protein 1), and Itch, are mainly expressed in neurons. In the current study, A20 expression was detected directly in the cytoplasm of rat neurons in the ischemia/reperfusion cortex for the first time. These spatial distribution characteristics suggest that neurons may play an important role in the mechanism by which A20 regulates inflammation in the brain. Although it is unclear why A20 is not upregulated in astrocytes and microglial cells, it is possible that there is no need for A20 in these cells since they are executors of the inflammatory response.

Studies investigating whether A20 exerts protective effects in the brain are contradictory. Some functional depletion studies found that CNS-specific A20 knockout mice exhibited activated astrocytes and microglia, increased inflammatory factor expression in the brain [37], and developed an aggravated form of autoimmune encephalomyelitis [38]. However, CNS-specific A20 KO failed to improve neurological outcome in a mouse MCAO model [35]. In contrast, a gain-of-function study demonstrated that overexpressing A20 in neurons significantly reduced hippocampal neuron apoptosis in a rat MCAO model [39]. The current study revealed that A20 overexpression could significantly inhibit neural function damage and reduce the volume of cerebral infarction after focal cerebral ischemia/reperfusion in SD rats after infection with lentivirus expressing the A20 gene. However, A20 silencing exerted the opposite effects. These results from two sides of A20 overexpression and silencing, respectively, indicated that A20 played a protective role at the early stage of ischemia/reperfusion in the brain, which suggests that altered A20 expression may help reduce brain injury. To further explain why neuronal A20 upregulation reduced focal cerebral ischemia/reperfusion-induced inflammatory injury, correlation analysis revealed that the number of A20-expressing neurons in the ischemia/reperfusion cortex was significantly positively correlated with neurological function score but negatively correlated with the cerebral infarction volume, glial cell activation, and pro-inflammatory cytokine content in the brain. This suggests that an increased number of A20-expressing neurons could alleviate inflammatory injury and suppress the glial cell inflammatory response. Therefore, these results suggest that neurons play an important role in regulating neuroinflammation by interaction with glial cells in response to inflammation. As such, upregulating neuronal A20 expression could be an important therapeutic strategy to reduce the inflammatory injury in the deficiency of endogenous A20 expression.

EA is an effective clinical treatment for ischemic stroke and inflammation-related diseases. Our previous study [51] demonstrated that EA could significantly inhibit early activation of the NF-kB signaling pathway after cerebral ischemia/reperfusion and reduce the subsequent inflammatory damage; however, the specific regulatory mechanism was not identified. In the current study, we not only confirmed the therapeutic effects of EA but also demonstrated that administration of EA beginning 2 h after cerebral ischemia could significantly increase A20 mRNA and protein levels in the brain. The A20 mRNA expression peaked earlier, and the mRNA and protein expression was prolonged compared with non-EA rats that underwent simple ischemia/reperfusion. More specifically, EA increased the number of neurons expressing A20 in the focal cerebral ischemia/reperfusion cortex. Taken together, these data suggest that EA could significantly change the temporal and spatial distribution of A20 in the cerebral ischemia/reperfusion area and confirmed that EA is an effective method to rapidly upregulate A20 expression. Furthermore, A20 silencing revealed that effects of EA on inhibiting the production of TNF-α and IL-1β, activating astrocytes and microglia, infarction formation, and improving neurological outcome were inhibited by blocking the expression of A20. Thus, A20 is an important regulatory target for EA for alleviating inflammatory injury after cerebral ischemia/reperfusion.

Moreover, the current study demonstrated that EA inhibited the phosphorylation of IKKβ and IkBα by increasing A20 expression. This reduced the nuclear translocation of NF-kB p65 and thereby blocked activation of the NF-kB signaling pathway. Accordingly, A20 silencing weakened the inhibitory effects of EA on NF-kB signaling. These results revealed that A20 was the key regulatory protein for EA to significantly inhibit the activation of NF-kB signaling. Since A20 expression was mainly upregulated in cortical neurons in the ischemic/reperfusion area and there was a close relationship between A20 and NF-kB signaling, this suggests that A20-expresssing neurons may be the main target through which EA inhibits the inflammatory response after acute cerebral ischemia by regulating the neuronal NF-kB signaling pathway.

The NF-kB signaling pathway is strongly activated in ischemic brain tissue, including neurons and glial cells. Generally, activation of the NF-kB signaling pathway in microglia and astrocytes promotes the formation of inflammatory lesion in the CNS [77–80]. However, it is controversial whether the activation of NF-kB signaling pathway in neurons exerts beneficial or detrimental impacts on the neuronal apoptosis [81, 82]. The current results suggested that at early stage of focal cerebral ischemia/reperfusion, the activation of NF-kB signaling pathway in neurons was largely inhibited by the upregulation of neuronal A20 expression that resulted in the transcription of some pro-inflammatory genes such as TNF-α and IL-1β which were consequently limited. Because of the large number of neurons, inhibiting the production of TNF-α and IL-1β from neurons could effectively reduce their content in the brain tissue, which decreased the amplification effects of positive feedback mediated by NF-kB signaling pathway and thereby depressed the activation of glial cells. Finally, the global NF-kB signaling pathway was suppressed in the cerebral ischemia/reperfusion area. Therefore, suppressing NF-kB signaling in neurons at the early stage of cerebral ischemia/reperfusion could prevent amplification of the inflammatory cascade and promote neuroprotection. In addition, activation of NF-kB subunits also affected neuronal survival after cerebral ischemia. Herrmann et al. [83] found that mice lacking neuronal IKKβ, which is the upstream kinase in the classical NF-kB signaling pathway, exhibited significantly reduced neuronal injury after cerebral ischemia/reperfusion. Multiple studies [5, 8, 84] also demonstrated that the nuclear translocation of NF-kB p65 played a central role in neuronal cell death after cerebral ischemia. Other reports demonstrated that there was a trend toward increased p65 and decreased c-Rel expression in the ischemic brain tissue of MCAO mice and oxygen glucose deprived neurons [5, 85]. The authors proposed that this is a key mechanism in ischemic brain injury. However, the current study revealed that upregulated neuronal A20 expression was likely the key mechanism by which early EA treatment inhibits IKKβ phosphorylation and reduces the neuronal nuclear translocation of p65 to prevent NF-kB signaling-induced inflammatory injury.

## Conclusions

In summary, the current results suggest that ischemia/reperfusion cortical neurons are the main cell types that express A20 and that there is a correlation between neuronal A20 expression and the neuroprotective effects. EA could upregulate neuronal A20 expression, which is essential for the anti-inflammatory effects of EA by suppressing neuronal NF-kB signaling in the brain of MCAO rats.

## Abbreviations

A1: Bcl-2 related protein A1α
A20: Tumor necrosis factor alpha-induced protein 3
CNS: Central nervous system
EA: Electroacupuncture
ELISA: Enzyme-linked immunosorbent assay
GFAP: Glial fibrillary acidic protein
Iba-1: Ionized calcium binding adapter molecule 1
IKKβ: IkB kinase beta
IL-1β: Interleukin-1 beta
IkBα: NF-kB inhibitor protein alpha
MCAO: Middle cerebral artery occlusion
NF-kB: Nuclear transcription factor kappa B
RT-qPCR: Real-time quantitative polymerase chain reaction
TNF-α: Tumor necrosis factor alpha
TTC: 2,3,5-Triphenyltetrazolium chloride

## References

[1] Mozaffarian D, Benjamin EJ, Go AS, Arnett DK, Blaha MJ, Cushman M (2016). Heart disease and stroke statistics—2016 update. Circulation.

[2] Liu L, Wang D, Wong KS, Wang Y (2011). Stroke and stroke care in China: huge burden, significant workload, and a national priority. Stroke.

[3] Abou-Chebl A (2013). Management of acute ischemic stroke. Curr Cardiol Rep.

[4] Luan H, Kan Z, Yong X, Lv C, Jiang W (2013). Rosmarinic acid protects against experimental diabetes with cerebral ischemia: relation to inflammation response. J Neuroinflammation.

[5] Inta I, Paxian S, Maegele I, Zhang W, Pizzi M, Spano P (2006). Bim and Noxa are candidates to mediate the deleterious effect of the NF-kappa B subunit RelA in cerebral ischemia. J Neurosci.

[6] Pizzi M, Sarnico I, Boroni F, Benetti A, Benarese M, Spano PF (2005). Inhibition of IkappaBalpha phosphorylation prevents glutamate-induced NF-kappaB activation and neuronal cell death. Acta Neurochir Supplement.

[7] Sarnico I, Lanzillotta A, Benarese M, Alghisi M, Baiguera C, Battistin L (2009). NF-kappaB dimers in the regulation of neuronal survival. Int Rev Neurobiol.

[8] Sarnico I, Lanzillotta A, Boroni F, Benarese M, Alghisi M, Schwaninger M (2009). NF-kappaB p50/RelA and c-Rel-containing dimers: opposite regulators of neuron vulnerability to ischaemia. J Neurochem.

[9] Sara Anna B, Giulia FT, Daniela U, Mery M, Laura B, Cristina L (2011). Nuclear factor kB-dependent neurite remodeling is mediated by Notch pathway. J Neuro sci.

[10] Nijboer CH, Heijnen CJ, Groenendaal F, van Bel F, Kavelaars A (2009). Alternate pathways preserve tumor necrosis factor-alpha production after nuclear factor-kappaB inhibition in neonatal cerebral hypoxia-ischemia. Stroke.

[11] Hayden MS, Ghosh S (2008). Shared principles in NF-kB signaling. Cell.

[12] Rius J, Guma M, Schachtrup C, Akassoglou K, Zinkernagel AS, Nizet V (2008). NF-kB links innate immunity to the hypoxic response through transcriptional regulation of HIF-1α. Nature.

[13] Candelario-Jalil E, Yang Y, Rosenberg GA (2009). Diverse roles of matrix metalloproteinase and tissue inhibitors of metalloproteinases in neuroinflammation and cerebral ischemia neuroscience. Neurosci.

[14] Vahedi K, Hofmeijer J, Juettler E, Vicaut E, George B, Algra A (2007). Early decompressive surgery in malignant infarction of the middle cerebral artery: a pooled analysis of three randomised controlled trials. Lancet Neurol.

[15] Iadecola C, Anrather J (2011). The immunology of stroke: from mechanisms to translation. Nature medicine.

[16] Dixit VM, Green S, Sarma V, Holzman LB, Wolf FW, O’Rourke K (1990). Tumor necrosis factor-alpha induction of novel gene products in human endothelial cells including a macrophage-specific chemotaxin. J Biol Chem.

[17] Opipari AW, Boguski MS, Dixit VM (1990). The A20 cDNA induced by tumor necrosis factor alpha encodes a novel type of zinc finger protein. J Biol Chem.

[18] Verstrepen L, Verhelst K, van Loo G, Carpentier I, Ley SC, Beyaert R (2010). Expression, biological activities and mechanisms of action of A20 (TNFAIP3). Biochem Pharmacol.

[19] Skaug B, Chen J, Fenghe D, He J, Ma A, Zhijian J (2011). Direct, noncatalytic mechanism of IKK inhibition by A20. Mol Cell.

[20] Shembade N, Ma A, Harhaj EW (2010). Inhibition of NF-kappaB signaling by A20 through disruption of ubiquitin enzyme complexes. Science.

[21] Wertz IE, O'Rourke KM, Zhou H, Eby M, Aravind L, Seshagiri S, Wu P (2004). De-ubiquitination and ubiquitin ligase domains of A20 downregulate NF-kappaB signalling. Nature.

[22] Wertz IE, Newton K, Seshasayee D, Kusam S, Lam C, Zhang J, Popovych N (2015). Phosphorylation and linear ubiquitin direct A20 inhibition of inflammation. Nature.

[23] Coornaert B, Carpentier I, Beyaert R (2009). A20: central gatekeeper in inflammation and immunity. J Biol Chem.

[24] Vereecke L, Beyaert R, van Loo G (2009). The ubiquitin-editing enzyme A20 (TNFAIP3) is a central regulator of immunopathology. Trends Immunol.

[25] Catrysse L, Vereecke L, Beyaert R, van Loo G (2014). A20 in inflammation and autoimmunity. Trends Immunol.

[26] Zammit NW, Grey ST (2014). Emerging roles for A20 in islet biology and pathology. Adv Exp Med Biol.

[27] Vereecke L, Beyaert R, van Loo G (2011). Genetic relationships between A20/TNFAIP3, chronic inflammation and autoimmune disease. Biochem Soc Trans.

[28] Parvatiyar K, Harhaj EW (2011). Regulation of inflammatory and antiviral signaling by A20. Microbes Infect.

[29] Gui J, Chen R, Xu W, Xiong S (2015). Remission of CVB3-induced myocarditis with Astragaloside IV treatment requires A20 (TNFAIP3) up-regulation. J Cell Mol Med.

[30] Kunter U, Daniel S, Arvelo MB, Choi J, Shukri T, Patel VI (2005). Combined expression of A1 and A20 achieves optimal protection of renal proximal tubular epithelial cells. Kidney Int.

[31] Ramsey HE, Da Silva CG, Longo CR, Csizmadia E, Studer P, Patel VI (2009). A20 protects mice from lethal liver ischemia/reperfusion injury by increasing peroxisome proliferator-activated receptor-alpha expression. Liver Transpl.

[32] Lutz J, le Luong A, Strobl M, Deng M, Huang H, Anton M (2008). The A20 gene protects kidneys from ischaemia/reperfusion injury by suppressing pro-inflammatory activation. J Mol Med.

[33] Li HL, Zhuo ML, Wang D, Wang AB, Cai H, Sun LH (2007). Targeted cardiac overexpression of A20 improves left ventricular performance and reduces compensatory hypertrophy after myocardial infarction. Circulation.

[34] Bi BL, Wang HJ, Bian H, Tian ZT (2015). Identification of therapeutic targets of ischemic stroke with DNA microarray. Eur Rev Med Pharmacol Sci.

[35] Mc Guire C, Rahman M, Schwaninger M, Beyaert R, van Loo G (2013). The ubiquitin editing enzyme A20 (TNFAIP3) is upregulated during permanent middle cerebral artery occlusion but does not influence disease outcome. Cell Death Dis.

[36] Dan H, Fang W, Rui Z, Jie W, Kodithuwakku ND, Lan S (2016). Clematichinenoside protects blood brain barrier against ischemic stroke superimposed on systemic inflammatory challenges through up-regulating A20. Brain Behav Immun.

[37] Guedes RP, Csizmadia E, Moll HP, Ma A, Ferran C, Da SC (2014). A20 deficiency causes spontaneous neuroinflammation in mice. J Neuroinflammation.

[38] Wang X, Deckert M, Xuan NT, Nishanth G, Just S, Waisman A (2013). Astrocytic A20 ameliorates experimental autoimmune encephalomyelitis by inhibiting NF-kB and STAT1-dependent chemokine production in astrocytes. Acta Neuropathol.

[39] Miao HS, Yu LY, Hui GZ, Guo LH (2005). Antiapoptotic effect both in vivo and in vitro of A20 gene when transfected into rat hippocampal neurons. Acta Pharmacol Sin.

[40] Liu AJ, Li JH, Li HQ, Fu DL, Lu L, Bian ZX (2015). Electroacupuncture for acute ischemic stroke: a meta-analysis of randomized controlled trials. Am J Chin Med.

[41] Li Y, Wang Y, Zhang H, Wu P, Huang W (2015). The effect of acupuncture on the motor function and white matter microstructure in ischemic stroke patients. Evid Based Complement Alternat Med.

[42] Ratmansky M, Levy A, Messinger A, Birg A, Front L, Treger I (2016). The effects of acupuncture on cerebral blood flow in post-stroke patients: a randomized controlled trial. J Altern Complement Med.

[43] Pan S, Zhan X, Su X, Guo L, Lv L, Su B (2011). Proteomic analysis of serum proteins in acute ischemic stroke patients treated with acupuncture. Exp Biol Med (Maywood).

[44] Yang EJ, Jiang JH, Lee SM, Hwang HS, Lee MS, Choi SM (2010). Electroacupuncture reduces neuroinflammatory responses in symptomatic amyotrophic lateral sclerosis model. J Neuroimmunol.

[45] Kim HW, Kang SY, Yoon SY, Roh DH, Kwon YB, Han HJ (2007). Low-frequency electroacupuncture suppresses zymosan-induced peripheral inflammation via activation of sympathetic post-ganglionic neurons. Brain Res.

[46] Zijlstra FJ, van den Berg-de Lange I, Huygen FJ, Klein J (2003). Anti-inflammatory actions of acupuncture. Mediators Inflamm.

[47] Gu G, Zhang Z, Wang G, Han F, Han L, Wang K (2011). Effects of electroacupuncture pretreatment on inflammatory response and acute kidney injury in endotoxaemic rats. J Int Med Res.

[48] Kavoussi B, Ross BE (2007). The neuroimmune basis of anti-inflammatory acupuncture. Integr Cancer Ther.

[49] Choi DC, Lee JY, Lim EJ, Baik HH, Oh TH (2012). Inhibition of ROS-induced p38MAPK and ERK activation in microglia by acupuncture relieves neuropathic pain after spinal cord injury in rats. Exp Neurol.

[50] Fu X, Wang YQ, Wang J, Yu J, Wu GC (2007). Changes in expression of nociceptin/orphanin FQ and its receptor in spinal dorsal horn during electroacupuncture treatment for peripheral inflammatory pain in rats. Peptides.

[51] Qin WY, Luo Y, Chen L, Tao T, Li Y, Cai YL (2013). Electroacupuncture could regulate the NF-kappaB signaling pathway to ameliorate the inflammatory injury in focal cerebral ischemia/reperfusion model rats. Evid Based Complement Alternat Med.

[52] Longa EZ, Weinstein PR, Carlson S, Cummins R (1989). Reversible middle cerebral artery occlusion without craniectomy in rats. Stroke.

[53] Buchan A, Pulsinelli WA (1990). Hypothermia but not the N-methyl-D-aspartate antagonist, MK-801, attenuates neuronal damage in gerbils subjected to transient global ischemia. J Neurosci.

[54] Busto R, Dietrich WD, Globus MY, Valdes I, Scheinberg P, Ginsberg MD (1987). Small differences in intraischemic brain temperature critically determine the extent of ischemic neuronal injury. J Cereb Blood Flow Metab.

[55] Garcia JH, Wagner S, Liu KF, Hu XJ (1995). Neurological deficit and extent of neuronal necrosis attributable to middle cerebral artery occlusion in rats. Statistical validation. Stroke.

[56] Ashwal S, Tone B, Tian HR, Cole DJ, Pearce WJ (1998). Core and penumbral nitric oxide synthase activity during cerebral ischemia and reperfusion. Stroke.

[57] Qiang W, Xingchun G, Lize X, Weilin J, Shaoyang C, Lichao H (2008). Trans-activator of transcription-mediated delivery of NEP1-40 protein into brain has a neuroprotective effect against focal cerebral ischemic injury via inhibition of neuronal apoptosis. Anesthesiology.

[58] Saito K, Suyama K, Nishida K, Sei Y, Basile AS (1996). Early increases in TNF-alpha, IL-6 and IL-1 beta levels following transient cerebral ischemia in gerbil brain. Neurosci lett.

[59] Kreutzberg GW (1996). Microglia: a sensor for pathological events in the CNS. Trends Neurosci.

[60] Cinthia F, Francesca A, Edgar M (2007). Astrocytes are active players in cerebral innate immunity. Trends Immunol.

[61] Barreto G, White RE, Ouyang Y, Xu L, Giffard RG (2011). Astrocytes: targets for neuroprotection in stroke. Cent Nerv Syst Agents Med Chem.

[62] Nowicka D, Rogozinska K, Aleksy M, Witte OW, Skangiel-Kramska J (2008). Spatiotemporal dynamics of astroglial and microglial responses after photothrombotic stroke in the rat brain. Acta Neurobiol Exp (Wars).

[63] Eng LF, Ghirnikar RS (1994). GFAP and astrogliosis. Brain Pathol.

[64] He J, Crews FT (2008). Increased MCP-1 and microglia in various regions of the human alcoholic brain. Exp Neurol.

[65] Wang Q, Wang F, Li X, Yang Q, Li X, Xu N (2012). Electroacupuncture pretreatment attenuates cerebral ischemic injury through α7 nicotinic acetylcholine receptor-mediated inhibition of high-mobility group box 1 release in rats. J Neuroinflammation.

[66] Young W, Rappaport ZH, Chalif DJ, Flamm ES (1987). Regional brain sodium, potassium, and water changes in the rat middle cerebral artery occlusion model of ischemia. Stroke.

[67] Stephenson D, Yin T, Smalstig EB, Hsu MA, Panetta J, Little S (2000). Transcription factor nuclear factor-kappa B is activated in neurons after focal cerebral ischemia. J Cereb Blood Flow Metab.

[68] Wang L, Zhang X, Liu L, Yang R, Cui L, Li M (2010). Atorvastatin protects rat brains against permanent focal ischemia and downregulates HMGB1, HMGB1 receptors (RAGE and TLR4), NF-kappaB expression. Neurosci lett.

[69] Garg P, Duncan RS, Kaja S, Koulen P (2010). Intracellular mechanisms of N-acylethanolamine-mediated neuroprotection in a rat model of stroke. Neurosci.

[70] Chen B, Liao WQ, Xu N, Xu H, Wen JY, Yu CA (2009). Adiponectin protects against cerebral ischemia-reperfusion injury through anti-inflammatory action. Brain res.

[71] Cho S, Park EM, Febbraio M, Anrather J, Park L, Racchumi G (2005). The class B scavenger receptor CD36 mediates free radical production and tissue injury in cerebral ischemia. J Neurosci.

[72] Kunz A, Abe T, Hochrainer K, Shimamura M, Anrather J, Racchumi G (2008). Nuclear factor-kappaB activation and postischemic inflammation are suppressed in CD36-null mice after middle cerebral artery occlusion. J Neurosci.

[73] Wang G, Guo Q, Hossain M, Fazio V, Zeynalov E, Janigro D (2009). Bone marrow-derived cells are the major source of MMP-9 contributing to blood–brain barrier dysfunction and infarct formation after ischemic stroke in mice. Brain res.

[74] Wang Q, van Hoecke M, Tang XN, Lee H, Zheng Z, Swanson RA (2009). Pyruvate protects against experimental stroke via an anti-inflammatory mechanism. Neurobiol Dis.

[75] Rock KL, Latz E, Ontiveros F, Kono H (2010). The sterile inflammatory response. Annu Rev Immunol.

[76] Pranski EL, Van Sanford CD, Dalal NV, Orr AL, Dipan K (2012). Comparative distribution of protein components of the A20 ubiquitin-editing complex in normal human brain. Neurosci lett.

[77] Dvoriantchikova G, Barakat D, Brambilla R, Agudelo C, Hernandez E, Bethea JR (2009). Inactivation of astroglial NF-kappa B promotes survival of retinal neurons following ischemic injury. Eur J Neurosci.

[78] Gao S, Mo J, Chen L, Wang Y, Mao X, Shi Y (2016). Astrocyte GGTI-mediated Rac1 prenylation upregulates NF-kappaB expression and promotes neuronal apoptosis following hypoxia/ischemia. Neuropharmacology.

[79] Michelle L, Block LZ, Jau-Shyong H (2007). Microglia-mediated neurotoxicity: uncovering the molecular mechanisms. Nat Rev Neurosci.

[80] Kaushal V, Schlichter LC (2008). Mechanisms of microglia-mediated neurotoxicity in a new model of the stroke penumbra. J Neurosci.

[81] Bhakar AL, Tannis LL, Zeindler C, Russo MP, Jobin C, Park DS (2002). Constitutive nuclear factor-kappa B activity is required for central neuron survival. J Neurosci.

[82] Zhang W, Potrovita I, Tarabin V, Herrmann O, Beer V, Weih F (2005). Neuronal activation of NF-kappaB contributes to cell death in cerebral ischemia. J Cereb Blood Flow Metab.

[83] Herrmann O, Baumann B, de Lorenzi R, Muhammad S, Zhang W, Kleesiek J (2005). IKK mediates ischemia-induced neuronal death. Nat Med.

[84] Ingrassia R, Lanzillotta A, Sarnico I, Benarese M, Blasi F, Borgese L (2012). 1B/(−)IRE DMT1 expression during brain ischemia contributes to cell death mediated by NF-kB/RelA acetylation at Lys310. Plos One.

[85] Crack PJ, Taylor JM, Ali U, Mansell A, Hertzog PJ (2006). Potential contribution of NF-kB in neuronal cell death in the glutathione peroxidase-1 knockout mouse in response to ischemia-reperfusion injury. Stroke.

